# Associations between metabolic syndrome and allergic diseases a nationwide study in Korea and literature review

**DOI:** 10.1038/s41598-026-41559-3

**Published:** 2026-03-03

**Authors:** Min Jung Kwon, Jihye An, Jin Woo Yu, Jung Eun Kim, Youin Bae, Young Lip Park, Jong Youn Moon, Sul Hee Lee

**Affiliations:** 1 Department of Dermatology, Soonchunhyang University Bucheon Hospital, Soonchunhyang University College of Medicine, Bucheon, Republic of Korea; 2 Department of Epidemic Intelligence Service, Incheon Communicable Diseases Center, Incheon, Republic of Korea; 3 Department of Dermatology, Soonchunhyang University Cheonan Hospital, Soonchunhyang University College of Medicine, Cheonan, Republic of Korea; 4 Department of Dermatology, Soonchunhyang University Seoul Hospital, Soonchunhyang University College of Medicine, Seoul, Republic of Korea; 5 Department of Preventive Medicine, Gachon University College of Medicine, Incheon, Republic of Korea; 6 Center for Public Health, Gachon University Gil Medical Center, Incheon, Republic of Korea

**Keywords:** Diseases, Health care, Immunology, Medical research, Risk factors

## Abstract

Atopic dermatitis (AD), allergic rhinitis (AR), and asthma are typical allergic diseases, with a common pathogenesis involving increased type 2 immunity. Despite numerous studies, the association between these diseases and metabolic syndrome (MetS) remains controversial. This study aimed to explore relationships between AD, AR, and asthma both to MetS and to each of its components. Population-based, cross-sectional data were obtained from the Korean National Health and Nutrition Examination Survey from 2019 to 2020. Multiple logistic regression analyses were conducted to evaluate the association between allergic diseases and MetS. The results were compared with those of previous studies of the association between MetS and allergic diseases, identified in a literature review following a search of PubMed and the Cochrane Library. Based on assessments of 1,329, 4,824, and 1,172 patients with AD, AR, and asthma, respectively, negative associations between both AD and AR and MetS were determined, after adjusting for confounding factors. By contrast, asthma was positively associated with MetS (odds ratio [OR] 0.58, 95% confidence interval [CI] 0.50–0.67; OR 0.73, 95% CI 0.67–0.78; OR 1.70, 95% CI 1.51–1.92, respectively). The heterogeneous findings highlight the need for further investigation of associations between the studied allergic diseases and MetS as well as each of its components.

**Supplementary Information:**

The online version contains supplementary material available at 10.1038/s41598-026-41559-3.

## Introduction

The increasing prevalence of metabolic syndrome (MetS) worldwide has led to its recognition as a global health concern and to studies of its association with various inflammatory diseases^1–3^, especially allergic diseases. Atopic dermatitis (AD), allergic rhinitis (AR), and asthma are common atopic or allergic diseases that share a pathophysiologic mechanism involving increased type 2 immunity. Several studies investigated the relationship between these three diseases and MetS, but their results were inconclusive. Thus, in this study the associations between AD, AR, and asthma and both MetS and its components were investigated using a population-based nationwide cross-sectional health survey. Further insights into the association between MetS and these allergic diseases were obtained in a literature review. The results of those studies were compared with our own.

## Materials and methods

### Study population

Data were obtained from the Korea National Health and Nutrition Examination Survey (KNHANES), conducted by the Korea Disease Control and Prevention Agency (KDCA). The KNHANES adopted a multi-stage, stratified, probability-clustered sampling method based on age, sex, and geographical location. The target population of the survey was the non-institutionalized civilian population of South Korea. The survey was conducted annually by trained professionals, including nutritionists, nurses, and individuals with degrees in public health. The data were statistically analyzed to guide health-related policies in South Korea and to support research on the risk associated with various diseases. The survey was composed of four main components: a health interview, a health behavior survey, a health examination, and a nutrition survey. The data can be downloaded from the KNHANES website (https://knhanes.kdca.go.kr/) and used for academic research purposes. The study design adhered to the principles outlined in the Declaration of Helsinki for research involving human subjects, and all survey participants provided written informed consent.

In the present study, data from KNHANES Ⅷ 2019 to 2020 were used to explore the relationship between the presence of allergic diseases (AD, AR, and asthma) and MetS. Participants ≥ 19 years of age who completed the questionnaires on the presence of allergic diseases and MetS were included. This study was approved by the IRB of Soonchunhyang University Bucheon Hospital (approval number SCHBC 2022-11-024).

### Survey components on allergic diseases and MetS

#### Definition of allergic diseases

Participants who answered “yes” to, “Have you ever been diagnosed with atopic dermatitis by a physician?” were assigned to the AD group, those who answered “yes” to, “Have you ever been diagnosed with allergic rhinitis by a physician?” to the AR group, and those who answered “yes” to, “Have you ever been diagnosed with asthma by a physician?” to the asthma group.

#### Definition of MetS

MetS was defined based on the revised criteria of the National Cholesterol Education Program (NCEP) Adult Treatment Panel III (ATP III)^1^, according to which the presence of three or more of the following five components constitute a diagnosis of MetS: (i) waist circumference (WC): ≥ 90 cm in men and ≥ 80 cm in women (in accordance with the International Obesity Task Force criteria for the Asian-Pacific population); (ii) triglycerides ≥ 150 mg/dL; (iii) high-density lipoprotein (HDL) cholesterol < 40 mg/dL in men and < 50 mg/dL in women; (iv) blood pressure (BP) ≥ 130/85 mmHg or antihypertensive medications; and (v) fasting blood glucose ≥ 110 mg/dL (fasting blood glucose ≥ 100 mg/dL, revised in 2005) or antidiabetic medications. Hypertriglyceridemia was defined as a triglyceride level ≥ 150 mg/dL, in accordance with the NCEP-ATP III criteria. Individuals taking lipid-lowering medications but not meeting this threshold were not classified as having hypertriglyceridemia.

### Other covariates

Demographic variables included age, sex, area of residence, education level, income, and occupation status. The data were obtained through the health interviews. The area of residence was categorized by dividing the 16 districts of South Korea into two groups: (i) urban regions, including Seoul, Gyeonggi, Busan, Daegu, Incheon, Gwangju, Daejeon, and Ulsan, and (ii) rural regions, including Gangwon, Chungbuk, Chungnam, Jeonnam, Jeonbuk, Gyeongbuk, Gyeongnam, and Jeju. Education level was classified into three categories: elementary school or lower, middle-school graduate and high-school graduate, or college graduate or higher. Income status was quantified by quartile. Occupation status was categorized as retired or working. Other covariates analyzed in this study included health-related behaviors such as physical activity, smoking history, alcohol consumption, and cancer history. Physical activity was categorized as vigorous activity (more than three times per week), moderate-intensity activity (more than five times per week), and others. Smoking history was categorized as ever and never. Alcohol consumption was classified into the following categories: none in the past year, < once/month, once/month, 2–4 times/month, 2–3 times/week, and ≥ 4 times/week. Detailed baseline characteristics are summarized in Supplementary Table 1.

### Statistical analysis

Statistical analyses were conducted using the Self-Assessment Survey (SAS) survey (ver. 9.4; SAS Institute, Inc., Cary, NC, USA), to account for the complex sampling design and sampling weights. The demographic data of the participants are presented as either the mean and standard error (SE) or the proportion and SE. Data from participants in the AD group were compared using the Rao-Scott χ2 test (for categorical variables) and an analysis of variance (for continuous variables). The association between allergic diseases and MetS was evaluated in multiple logistic regression analyses. Adjustments were made for age, sex, monthly income, region of residency, education level, smoking status, drinking habits, and cancers that could significantly affect MetS. Possible associations between allergic diseases and MetS are expressed as odds ratios (ORs) and 95% confidence intervals (CIs). In all analyses, p-values were two-tailed, and a P-value < 0.05 was considered to indicate statistical significance. Adjustments were made for age, sex, area of residence, education level, income, occupation status, physical activity, smoking history, alcohol consumption, and cancer history.

## Results

Among the 47,144 patients included in this study, 1,329 had been diagnosed with AD, 4,824 with AR, and 1,172 with asthma. The demographic characteristics of the participants are shown in Table 1. There were significant differences in age between participants with allergic diseases and the control group. Those with AD and AR were significantly younger (*P* < 0.001) than participants in the control group whereas those with asthma were significantly older (*P* < 0.001). In terms of sex, significant differences from the control group were determined for the AR and asthma groups (*P* < 0.001), but not for the AD group. Specifically, the prevalence of males in the asthma group was twice as high as that in the control group. Compared to the control group, participants with AD and AR were more educated (*P* < 0.001) whereas those with asthma were less educated (*P* < 0.001) than controls.

**Table 1:** Factors associated with atopic dermatitis, allergic rhinitis, and asthma. *OR* odds ratio, *CI* confidence interval.

Variables	Atopic dermatitis			Allergic rhinitis			Asthma		
	Adjusted OR	95% CI	*P* value	Adjusted OR	95% CI	*P* value	Adjusted OR	95% CI	*P* value
Metabolic syndrome			< 0.0001			< 0.0001			< 0.0001
No	1.00			1.00			1.00		
Yes	0.58	(0.50–0.67)		0.73	(0.67–0.78)		1.70	(1.51–1.92)	
Sex			0.7090			< 0.0001			< 0.0001
Female	1.00			1.00			1.00		
Male	0.98	(0.86–1.11)		0.72	(0.67–0.77)		2.42	(2.10–2.79)	
Age			< 0.0001			< 0.0001			< 0.0001
19–64	1.00			1.00			1.00		
65-	0.50	(0.40–0.63)		0.53	(0.47–0.60)		3.98	(3.40–4.65)	
Region			0.0518			0.6037			0.9724
Rural	1.00			1.00			1.00		
Urban	1.12	(1.00–1.26)		1.02	(0.95–1.09)		1.00	(0.89–1.13)	
Education level			< 0.0001			< 0.0001			< 0.0001
Elementary school graduate or lower	1.00			1.00			1.00		
Middle school graduate	1.12	(0.82–1.54)		1.19	(1.02–1.40)		1.11	(0.93–1.32)	
High school graduate, or college graduate or higher	2.04	(1.61–2.59)		2.01	(1.77–2.27)		0.59	(0.50–0.70)	
Income			0.3271			0.0937			0.5572
Low	1.00			1.00			1.00		
High	0.94	(0.84–1.06)		0.94	(0.88–1.01)		1.04	(0.91–1.20)	
Occupation status			< 0.0001			0.2430			< 0.0001
Retired	1.00			1.00			1.00		
Working	1.48	(1.32–1.67)		1.04	(0.97–1.12)		1.46	(1.28–1.67)	
Physical activity			0.0157			< 0.0001			0.5885
Other	1.00			1.00			1.00		
Moderate intensity activity more than five times per week	1.19	(1.06–1.34)		4.66	(4.28–5.08)		1.07	(0.94–1.21)	
Vigorous activity more than three times per week	1.14	(0.95–1.38)		5.03	(4.51–5.61)		1.08	(0.84–1.38)	
Smoking			0.0121			0.0005			0.2838
No	1.00			1.00			1.00		
Yes (present)	0.83	(0.72–0.96)		1.18	(1.07–1.29)		1.09	(0.93–1.28)	
Drinking			0.0164			0.0237			< 0.0001
Not drinking for the last year	1.00			1.00			1.00		
Less than once a month	1.16	(0.96–1.40)		0.98	(0.89–1.08)		0.85	(0.72–1.02)	
Once a month	1.23	(1.00–1.53)		1.02	(0.91–1.15)		0.72	(0.57–0.91)	
Two to four times a month	1.29	(1.07–1.55)		1.07	(0.97–1.17)		0.67	(0.56–0.81)	
Two to three times a week	1.11	(0.90–1.37)		1.00	(0.90–1.12)		0.67	(0.54–0.81)	
More than four times a week	0.87	(0.65–1.17)		0.82	(0.70–0.96)		0.66	(0.53–0.83)	
Cancer			0.0359			0.2354			0.3588
No	1.00			1.00			1.00		
Yes	0.45	(0.21–0.95)		0.86	(0.66–1.11)		0.82	(0.53–1.26)	

In addition, participants in the AD group had higher rates of working status (*P* < 0.001) and vigorous physical activity (*P* = 0.016) than controls. The AD group also contained smaller proportions of smokers (*P* = 0.012), heavy drinkers (more than four times a week) (*P* = 0.016), and those with a history of cancer than controls.

Members of the AR group had a higher rate of vigorous physical activity (*P* < 0.001) and significantly lower rates of heavy drinking (*P* = 0.024) and smoking (*P* = 0.005) than the control group. In the asthma group, the rate of working status was higher and the rate of heavy drinking was lower than in the control group (both *P* < 0.001).

The analysis of the association between each allergic disease and age showed a significant association with ages 1 to 64 years and ≥ 65 years in the AD and AR groups. By contrast, no age-related association between asthma and other allergic diseases was determined.

The associations between MetS and the prevalence of each allergic disease were investigated in multivariate logistic regression analyses. After adjustments for potential confounding factors, the prevalence of MetS was negatively associated with both AD and AR (both *P* < 0.001) and positively associated with asthma (*p* < 0.001). In a subgroup analysis, the negative association between MetS and AD was still significant after adjustment for sex (male: OR 0.63, 95% CI 0.51–0.77, *P* < 0.0001; female: OR 0.58, 95% CI 0.46–0.73, *P* < 0.0001). There was no association between MetS and AD among older adults (≥ 65 years).

Table 2 shows the differences in MetS and its components according to the presence of allergic diseases. Participants in the AD group were less likely to have high BP and diabetes mellitus (DM) than those of the control group (OR 0.53, 95% CI 0.46–0.61, *P* < 0.001; OR 0.61, 95% CI 0.53–0.70, *P* < 0.001, respectively). Participants in the AR group were less likely to have low HDL-cholesterol, high BP, or DM (OR 0.86, 95% CI 0.80–0.92, *P* < 0.001; OR 0.60, 95% CI 0.56–0.64, *P* < 0.001; OR 0.71, 95% CI 0.66–0.77, *P* < 0.001; respectively). However, participants in the asthma group were more likely to have all components of MetS except high triglyceride (OR 0.73, 95% CI 0.64–0.84, *P* < 0.001).

**Table 2:** The relationship between atopic dermatitis, allergic rhinitis, asthma and metabolic syndrome, and a subgroup analysis. *OR* odds ratio, *CI* confidence interval.

Variables	Atopic dermatitis			Allergic rhinitis			Asthma		
	Adjusted OR	95% CI	*P* value	Adjusted OR	95% CI	*P* value	Adjusted OR	95% CI	*P* value
Waist circumference			0.2159			0.1007			0.0002
Men < 90 cm, women < 85 cm	1.00			1.00			1.00		
Men ≥ 90 cm, women ≥ 85 cm	0.92	(0.81–1.05)		1.06	(0.99–1.13)		1.27	(1.12–1.44)	
Triglycerides			0.0772			0.0008			< 0.0001
< 150 mg/dL	1.00			1.00			1.00		
≥ 150 mg/dL	0.88	(0.77–1.01)		0.88	(0.82–0.95)		0.73	(0.64–0.84)	
High density lipoprotein cholesterol			0.1143			< 0.0001			< 0.0001
Men > 40 mg/dL, women > 50 mg/dL	1.00			1.00			1.00		
Men ≤ 40 mg/dL, women ≤ 50 mg/dL	0.90	(0.80–1.03)		0.86	(0.80–0.92)		1.38	(1.22–1.57)	
Blood pressure			< 0.0001			< 0.0001			< 0.0001
SBP < 130 mmHg, DBP < 85 mmHg	1.00			1.00			1.00		
SBP ≥ 130 mmHg, DBP ≥ 85 mmHg	0.53	(0.46–0.61)		0.60	(0.56–0.64)		2.68	(2.35–3.06)	
Diabetes mellitus			< 0.0001			< 0.0001			< 0.0001
< 100 mg/dL	1.00			1.00			1.00		
≥ 100 mg/dL	0.61	(0.53–0.70)		0.71	(0.66–0.77)		2.38	(2.10–2.71)	

Following a literature review using PubMed and the Cochrane Library, performed to identify studies investigating the association between MetS and allergic diseases, 42 studies (23 for AD, 9 for AR, and 10 for asthma) were included for further analysis. Their results are discussed below and summarized in Tables 3 and 4, and 5.

**Table 3:** Studies of the association between each component of metabolic syndrome and atopic dermatitis. *AD* atopic dermatitis, *HDL* high-density lipoprotein, *ICD* International Classification of Diseases, *OR* odds ratio, *DM* diabetes mellitus, *HBP* high blood pressure, *MetS* metabolic syndrome, *BP* blood pressure, *LDL* low-density lipoprotein, *RR* risk ratio, *WC* waist circumference, *HR* hazard ratio, *SBP* systolic blood pressure, *DBP* diastolic blood pressure, *SNOMET-CT* Systematized Nomenclature of Medicine Clinical Terms, *CI* confidence interval.

Study	Study design	Location	Definition of AD	Ever/Current Diagnosis	Central obesity	Dyslipidemia	Hypertension	Hyperglycemia	Conclusion
Megna et al. (2017)^26^	Prospective	Italy	Physician’s diagnosis	Current		No significant difference between adult-onset AD and persistent AD	Hypertension was more frequent in adult-onset AD than in persistent AD (13.7 vs. 2.6%, *P* < 0.01)	No significant difference between adult-onset AD compared to persistent AD	Hypertension was more frequent than persistent AD in adult-onset AD
Schäfer et al. (2003)^22^	Prospective	Germany	Physician’s diagnosis	Ever		AD was associated with higher HDL-cholesterol (61.0 vs. 54.9 mg/dL, *P* < 0.01)			Positive association between HDL-cholesterol levels and AD
Uehara et al. (2002)^27^	Prospective	Japan	Hanifin and Rajka criteria	Ever			AD was associated with a lower incidence of hypertension		Lower incidence of hypertension in patients with AD
Roh et al. (2022)^16^	Cross-sectional	USA	ICD-10 codes	Ever		AD was associated with a higher incidence of dyslipidemia (OR 1.37, *P* < 0.001)	AD was associated with a higher incidence of hypertension (OR 1.27, *P* < 0.001)	AD was associated with a higher incidence of type 2 DM (OR 1.19, *P* < 0.001)	AD was associated with a higher incidence of dyslipidemia, hypertension, type2 DM and MetS
Richard et al. (2021)^17^	Cross-sectional	France	Self- reported physician’s diagnosis	Ever		Dyslipidemia was more prevalent in AD patients (11.6 vs. 9.45%)	HBP was more prevalent in AD patients (13.53 vs. 12.85%)		After adjustment for sex and age, HBP and dyslipidemia were more prevalent in AD patients
Jung et al. (2021)^18^	Cross-sectional	Korea	ICD-10 codes	Ever		Patients with AD had a significantly higher risk of hyperlipidemia (HR 33.02, *P* < 0.001)	Patients with AD had a significantly higher risk of hypertension (HR 4.86, *P* < 0.001)	Patients with AD had a significantly higher risk of type 2 DM (HR 2.96, *P* < 0.001)	Metabolic disease was significantly higher in AD patients than in the control group
Shalom et al. (2019)^4^	Cross-sectional	Israel	Physician’s diagnosis	Ever	Moderate and severe AD were associated with central obesity (22.2 vs. 18.6%, *P* < 0.001)	AD in the entire group of patients was associated with a higher prevalence of dyslipidemia (47.1 vs. 28.5%, *P* < 0.001)	Moderate and severe AD were associated with hypertension (27.9 vs. 15.3%, *P* < 0.001)	Moderate and severe AD were associated with DM (15.9 vs. 9.2%; *P* < 0.001)	Moderate and severe AD were associated with higher prevalence rates of MetS (17.0 vs. 9.4%) and its components (obesity: 22.2 vs. 18.6%; DM: 15.9 vs. 9.2%; hypertension 27.9 vs. 15.3%; dyslipidemia 47.1 vs. 28.5%, all *P* < 0.001)
Ivert et al. (2019)^19^	Cross-sectional	Sweden	ICD-8, 9, 10 codes	Ever		Hyperlipidemia was more prevalent in severe AD (6.0 vs. 5.0%, *P* < 0.001)	Hypertension was more prevalent in severe AD patients (21.0 vs. 16.4%, *P* < 0.001)	Hyperglycemia was more prevalent in severe AD patients (7.9 vs. 6.3%, *P* < 0.001)	DM, hyperlipidemia, and hypertension were more prevalent in severe AD patients than in controls
Silverberg et al. (2018)^23^	Cross-sectional	USA	Modified United Kingdom Working Party Criteria	Current			AD was associated with higher odds of high BP (OR 1.46, 95% CI 1.18–1.80)	AD was associated with higher odds of DM (OR 1.52, 95% CI 1.16–1.99)	AD was associated with higher odds of high BP and DM
Standl et al. (2017)^24^	Cross-sectional	Germany	ICD-10 codes	Current		AD was not associated with HDL-cholesterol, LDL-cholesterol, triglycerides or total cholesterol	AD increases the risk of hypertension (RR 1.04, 95% CI 1.02–1.06)		Marginally increased risk of hypertension in AD patients
Drucker et al. (2017)^28^	Cross-sectional	Canada	Self-reported physician’s diagnosis	Ever			AD was inversely associated with hypertension (OR 0.87, 95% CI 0.83–0.90)	AD was inversely associated with type 2 DM (OR 0.78, 95% CI 0.71–0.84)	AD was inversely associated with hypertension and type 2 DM
Lee et al. (2017)^6^	Cross-sectional	Korea	Self-reported physician’s diagnosis	Ever	Women with AD had central obesity more often (OR 1.73, 95% CI 1.09–2.75); the association was not found in men	Women with AD had hypertriglyceridemia more often (OR 2.20, 95% CI 1.19–4.05); the association was not found in men		Women with AD had hyperglycemia more often (OR 1.44, 95% CI 0. 65–3.16) (not significant); the association was not found in men	MetS and its components (central obesity and hypertriglyceridemia) correlated positively with the presence of AD in women
Lee et al. (2017)^15^	Cross-sectional	Korea	Self-reported physician’s diagnosis	Ever	Did not find any association between obesity and AD		Hypertension was not associated with AD (OR 0.94, 95% CI 0.75–1.17)	DM was inversely associated with adult AD (OR 0.55, 95% CI 0.39–0.78)	Inverse correlation between MetS and DM, hypertension, and AD
Park et al. (2017)^72^	Cross-sectional	Korea	Self-report	Ever				No association between impaired fasting glucose and AD or DM and AD	No significant association between fasting glucose and AD
Egeberg et al. (2017)^29^	Cross-sectional	Denmark	Danish version of ICD	Current			Prevalence of hypertension was lower in AD than in psoriasis patients (OR 0.58, 95% CI 0.54–0.63)	Prevalence of DM was lower in AD than in psoriasis patients (OR 0.47, 95% CI 0.42–0.52)	Prevalence of hypertension and DM was lower in AD patients
Lee et al. (2016)^5^	Cross-sectional	Korea	Self-reported physician’s diagnosis	Ever	High WC (≥ 80 cm) was associated with AD in women (OR 2.12, 95% CI 1.11–4.03); the association was not found in men				High WC was significantly associated with AD in women
Andersen et al. (2017)^31^	Cross-sectional	Denmark	Danish version of ICD	Ever				After adjustment for socioeconomic status, smoking, comorbidity, and medication use, the independent risk of type2 DM was significantly decreased in AD patients (adjusted HR 0.76, 95% CI 0.68–0.83)	Independent risk of type2 DM was significantly decreased in AD patients
Silverberg et al. (2015)^7^	Cross-sectional	USA	Hanifin and Rajka criteria	Current	High WC (≥ 85th percentile) was associated with AD (OR 3.92, 95% CI 1.50–10.26)		AD was associated with higher SBP (OR 2.96, 95% CI 1.16–7.51) and DBP (OR 2.72, 95% CI 1.01–7.36)		AD was associated with central obesity and increased BP
Silverberg and Greenland (2015)^20^	Cross-sectional	USA	Self-reported physician’s diagnosis	Ever		AD was associated with high cholesterol (OR 1.29, 95% CI 1.15–1.45)	AD was associated with hypertension (OR 1.48, 95% CI 1.18–1.85)		AD was associated with high cholesterol and increased BP
Kok et al. (2019)^8^	Retro-spective	Singapore	SNOMET-CT diagnoses codes	Current	Those who were obese were likely to have moderate-to-severe AD (OR 1.44, 95% CI 1.28–1.63, *P* < 0.001)	Hyperlipidemia was associated with moderate-to-severe AD (OR 1.43, 95% CI 1.11–1.84, *P* = 0.006)	Having hypertension was associated with having moderate-to-severe AD (OR 1.71, 95% CI 1.25–2.35, *P* = 0.001)	Having type 2 DM was strongly associated with an increased risk of moderate-to-severe AD (OR 5.62, 95% CI 2.15–14.6, *P* < 0.001)	Metabolic and atopic conditions were significantly correlated with having moderate-to-severe AD
Radtke et al. (2017)^9^	Retro-spective	Germany	ICD-10 codes	Current	Obesity was associated with AD (prevalence ratio 1.17, 95% CI 1.13–1.20)	AD was associated with a lower prevalence ratio of hyperlipidemia (prevalence ratio 0.94, 95% CI 0.91–0.95)	AD was associated with a lower prevalence ratio of hypertension (prevalence ratio 0.83, 95% CI 0.81–0.85)	AD was associated with a lower prevalence ratio of DM (prevalence ratio 0.82, 95% CI 0.80–0.85)	AD was associated with obesity but not with increased risk of hypertension, hyperlipidemia, or DM
Kwa and Silberberg (2017)^25^	Retro-spective	USA	ICD-10 codes	Ever			AD was related to hypertension (OR 1.19, 95% CI 1.17–1.21), and the association was stronger for women		AD was associated with hypertension
Augustin et al. (2015)^21^	Retro-spective	Germany	ICD-10 codes	Ever		AD was associated with hyperlipidemia (prevalence ratio 1.29, 95% CI 1.12–1.49)	No association between AD and hypertension (prevalence ratio 1.03, 95% CI 0.84–1.24)		AD was associated with hyperlipidemia, but not hypertension

**Table 4:** Studies of the association between each component of metabolic syndrome and allergic rhinitis. *AR* allergic rhinitis, *BP* blood pressure, *CI* confidence interval, *OR* odds ratio, *FPG* fasting plasma glucose, *MetS* metabolic syndrome, *SBP* systolic blood pressure, *DBP* diastolic blood pressure.

Study	Study design	Location	Definition of AR	Ever/Current Diagnosis	Central obesity	Dyslipidemia	Hypertension	Hyperglycemia	Conclusion
Ferrari et al. (2019)^32^	Cross-sectional	Italy	Self-reported physician’s diagnosis	Ever	Lower risk of rhinitis in overweight patients (relative risk ratio 0.86, 95% CI 0.79–0.94)		Rhinitis patients had high BP (Relative risk ratio 1.42, 95% CI 1.13–1.77)		Rhinitis patients were less obese but more likely to have hypertension
Han et al. (2016)^33^	Cross-sectional	USA	Self-reported physician’s diagnosis	Ever	In children, central obesity was associated with reduced odds of AR (adjusted OR 0.35; 95% CI 0.19–0.64; *P* < 0.01)				In children, central obesity was associated with reduced odds of AR, regardless of sex
Hwang et al. (2016)^38^	Cross-sectional	Korea	Self-reported physician’s diagnosis	Ever			AR prevalence was significantly lower in patients with high BP (OR 0.85, 95% CI 0.77–0.94)	AR prevalence was significantly lower in patients with impaired FPG (OR 0.81, 95% CI 0.73–0.89)	AR prevalence was significantly lower in patients with MetS (OR 0.84, 95% CI 0.76–0.93)
Hashimoto and Futamura (2010)^37^	Cross-sectional	Japan	Self-reported physician’s diagnosis	Ever			Rhinitis was associated negatively with SBP (*P* = 0.034)	Rhinitis was associated negatively with FPG (*P* = 0.0478)	Patients with higher levels of FPG and higher SBP had a lower prevalence of rhinitis
Heinrich J and Döring A (2004)^73^	Cross-sectional	Germany	Self-reported physician’s diagnosis	Ever			Neither average SBP (132.3 vs. 132.9 mmHg, *P* = 0.64) nor DBP (83.8 vs. 83.8 mmHg, *P* = 0.97) differed significantly between men with and without rhinitis Adjusted prevalence rate of hypertension was also not different between males with and without rhinitis (OR 1.02, 95% CI 0.728–1.436, *P* = 0.74)		No statistically significant associations between rhinitis and confounder-adjusted BP means or hypertension in men or women
Heinrich et al. (2003)^74^	Cross-sectional	Germany	Self-reported physician’s diagnosis	Ever			Average SBP (*P* = 0.17), DBP (*P* = 0.60) and adjusted prevalence rate (*P* = 0.25) were not significantly different between men with and without rhinitis		No statistically significant associations between rhinitis and BP in men or women
Saito et al. (2003)^35^	Cross-sectional	Japan	Physician’s diagnosis	Ever			No difference in SBP between patients with and without AR; DBP, however, was higher in patients without AR		SBP did not differ between patients with and without AR. DBP, however, was higher in subjects without AR
Kony et al. (2003)^36^	Cross-sectional	France	Self-reported physician’s diagnosis	Ever			Hypertension was more frequent in men with rhinitis than in men without rhinitis (35.7 vs. 15.6%, *P* = 0.005; OR 3.0, 95% CI 1.37–6.64), even after adjustment for potential confounding factors (OR 2.6, 95% CI 1.14–5.91)		Association between rhinitis and SBP was found in men, not in women
Sheha et al. (2021)^34^	Retrospective	Egypt	AR and its impact on asthma guidelines	Current		Serum lipid profiles in AR patients were significantly higher than in controls (*P* < 0.001) Total cholesterol correlated positively with AR severity and the quality of life			Dyslipidemia was associated with AR severity

**Table 5:** Studies of the association between each component of metabolic syndrome and asthma. *FEF* forced expiratory flow, *FVC* forced vital capacity, *FEV1* forced expiratory volume in 1 s, *WC* waist circumference, *DM* diabetes mellitus, *BP* blood pressure, *OR* odds ratio, *CI* confidence interval, *MetS* metabolic syndrome, *BMI* body mass index, *HDL* high density lipoprotein.

Study	Study design	Location	Definition of asthma	Ever/Current diagnosis	Central obesity	Dyslipidemia	Hypertension	Hyperglycemia	Conclusion
Nejatifar et al. (2022)^39^	Prospective	Iran	Physician’s diagnosis	Ever	FEF, FVC and FEV1 had a significant negative correlation with WC (*P* < 0.05)	FEF, FVC and FEV1 was lower in patients with hypertriglyceridemia (*P* < 0.05)	FEF, FVC and FEV1 were lower in patients with hypertension (*P* < 0.05)	FEF, FVC and FEV1 were lower in patients with DM (*P* < 0.05)	WC and cardiovascular risk factors (including DM, hypertriglyceridemia, hypertension) had a significantly negative correlation with spirometry indices (*P* < 0.05)
Brumpton et al. (2013)^40^	Prospective	Norway	Self-reported physician’s diagnosis	Ever	High WC (adjusted OR 1.62, 95% CI 1.36–1.94) was associated with an increased risk of incident asthma in adults	Hypertriglyceridemia was not associated with an increased risk of incident asthma in adults	Elevated BP was not associated with an increased risk of incident asthma in adults	Elevated glucose or DM (adjusted OR 1.43, 95% CI 1.01–2.04) was associated with an increased risk of incident asthma in adults	MetS was a risk factor for incident asthma (adjusted OR 1.57, 95% CI 1.31–1.87)
Tomita et al. (2019)^42^	Cross-sectional	Japan	Self-reported physician’s diagnosis	Ever	In women, BMI 25–29.9 kg/m^2^ or ≥ 30 kg/m^2^, WC ≥ 90 cm, and waist-to-height ratio ≥ 0.5 were shown to be significant risk factors for asthma, with adjusted OR (95% CI) of 1.92 (1.35–2.75), 2.24 (1.23–4.09), 1.89 (1.30–2.75), and 1.53 (1.15–2.03), respectively				Obesity measures were shown to be significant risk factors for late-onset asthma but only in middle-aged Japanese women, not in middle-aged Japanese men.
Park et al. (2018)^41^	Cross-sectional	Korea	Self-reported physician’s diagnosis	Ever	Patients with a larger WC were especially likely to have asthma (adjusted OR 1.27, 95% CI 1.14–1.41)	Patients with a lower HDL-cholesterol were more likely to have asthma (adjusted OR 0.87, 95% CI 0.79–0.95)			Patients with MetS had a significantly higher prevalence of asthma (adjusted OR 1.34; 95% CI 1.09–1.64)
Demoly et al. (2010)^43^	Cross-sectional	France, Germany, Italy, Spain and the UK	Self-reported physician’s diagnosis	Ever	In patients whose asthma was not well-controlled, a significantly higher proportion were obese than among patients who had at least well-controlled asthma (30.4 vs. 23.3%, *P* < 0.001)				A higher proportion of patients with not well-controlled asthma were obese compared to patients who had well-controlled asthma
Zhang et al. (2009)^75^	Cross-sectional	Canada	Self-reported physician’s diagnosis	Current			High BP correlated positively with asthma (adjusted OR 1.39, 95% CI 1.27–1.53)		High BP has positive correlation with asthma
Adams et al. (2006)^76^	Cross-sectional	Australia	Self-reported physician’s diagnosis	Current				DM was not associated with asthma (aged > 55)	DM was not associated with asthma(aged > 55)
Nystad et al. (2004)^44^	Cross-sectional	Norway	Self-reported physician’s diagnosis	Ever	Compared with people with a BMI < 25, overweight (BMI: 25–29) men and women had relative risks of asthma of 1.27 (95% CI 1.13–1.43) and 1.30 (95% CI 1.17–1.45), respectively, while obese (BMI ≥ 30) men and women had relative risks of 1.78 (95% CI 1.35–2.34) and 1.99 (95% CI 1.67–2.37), respectively				Overweight or obese persons reported asthma more often than did thinner people, after adjustment for smoking, education, and physical activity
Chen et al. (2002)^45^	Cross-sectional	Canada	Self-reported physician’s diagnosis	Ever	The adjusted OR for women with a BMI of at least 30.0 kg/m^2^ versus 20.0–24.9 kg/m^2^ was 1.9 (95% CI 1.1–3.4); the corresponding OR of 1.1 (95% CI 0.3–3.6) for men was not significantly different				Obesity was related to asthma development in women but not in men
Enright et al. (1996)^77^	Cross-sectional	USA	Physician’s diagnosis	Ever		Controlling for smoking status, age, gender, and diagnoses of chronic bronchitis and emphysema, higher levels of HDL-cholesterol were significantly associated with asthma			Asthma was associated with a higher HDL-cholesterol

### AD and metabolic syndrome

The 23 studies from various countries and ethnicities included of prospective, cross-sectional, and retrospective designs. A substantial majority (17 out of 23) found a positive association between AD and MetS, but five studies reported a negative correlation. The outcomes regarding each component of MetS were also heterogeneous.

### AD and central obesity

A positive relationship between central obesity and AD^4–9^ was reported in six of the seven studies. The exception was the cross-sectional study by Lee et al.^6^, conducted in Korea, in which no clear association was determined. However, the authors showed that women, but not men, with AD were more likely to have central obesity (OR 1.73, 95% CI 1.09–2.75). Similarly, another cross-sectional study from Korea^5^ found that a WC ≥ 80 cm was associated with AD in women (OR 2.12, 95% CI 1.11–4.03), but not in men. The authors suggested that this difference is due to the higher level of estrogen produced by adipose tissue in women, which in turn, could result in eosinophilic granulation, eosinophilic adhesion to human mucosal cells, and enhanced leukocyte survival, all of which contribute to an increase in chronic inflammation^10–14^.

### AD and dyslipidemia

The relationship between AD and dyslipidemia was investigated in 13 studies, with the majority (9 out of 13) reporting a positive correlation^4,8,15–21^. In the cross-sectional study of a Korean population by Lee et al.^6^, women with AD had a higher probability of hypertriglyceridemia (OR 2.20, 95% CI 1.19–4.05), whereas there was no similar association in men. However, Radtke et al.^9^ analyzed German statutory health insurance data and found that AD was associated with a lower prevalence ratio of hyperlipidemia (prevalence ratio 0.94, 95% CI 0.91–0.95). In the study by Schäfer et al.^22^, based on the third MONICA (MONItoring of trends and determinants in CArdiovascular disease) data, a significantly higher concentration of HDL cholesterol was determined in the population with AD than in controls (61.0 vs. 54.9 mg/dL; *P* < 0.01).

### AD and hypertension

A positive association was determined in 12 of the 18 studies investigating the association between AD and hypertension^4,7,8,16–20,23–26^. A longitudinal analysis of adult German National Health Insurance beneficiaries by Standl etal^24^. demonstrated a dose-response relationship between AD and hypertension, with the most pronounced effect occurring in individuals with AD requiring systemic treatment. However, four studies, from Japan, Canada, Denmark, and Germany, reported an inverse correlation between the prevalence of hypertension and AD^9,27–29^.

### AD and hyperglycemia

In a review of 14 studies examining the association between AD and hyperglycemia, the findings were unclear, with seven studies reporting a positive correlation^4,8,15,16,18,19,23^. The above-cited Korean study of Lee et al.^6^ also showed that women, but not men, with AD were more likely to have hyperglycemia (OR 1.44, 95% CI 0.65–3.16). The authors attributed their findings to the higher estrogen levels in women, given that estrogen inhibits the production of cortisol^30^. Four cross-sectional studies, conducted in Canada, Korea, and Denmark^15,28,29,31^, along with a retrospective study in Germany^9^ reported an inverse correlation between hyperglycemia and AD.

### AR and MetS

A review of the nine studies examining the association between AR and MetS showed a consistent association of central obesity, dyslipidemia, and hyperglycemia, but heterogeneity regarding hypertension.

Ferrari et al.’s study^32^ of a population in Italy found a lower risk of rhinitis in overweight individuals (relative risk ratio 0.86, 95% CI 0.79–0.94). A cross-sectional study in the USA likewise demonstrated reduced odds of AR in children with central obesity (adjusted OR 0.35, 95% CI 0.19–0.64)^33^.

In the retrospective case-control study by Sheha et al.^34^, the levels of total cholesterol (TC) and low-density lipoprotein (LDL) cholesterol were significantly higher in AR patients than in controls (mean ± standard deviation [SD] 225.65 ± 62.91 mg/dL and 187.92 ± 35.24 mg/dL, respectively; and mean ± SD of LDL cholesterol 151.57 ± 60.42 mg/dL and 121.57 ± 19.03 mg/dL, respectively; *P* < 0.001). The authors also identified a positive correlation between TC and the severity of AR, measured by the visual analog scale (Pearson correlation coefficient 0.31; *P* = 0.039).

Studies of the association between AR and hypertension produced conflicting results. Saito et al.^35^, Ferrari et al.^32^, and Kony etal^36^. identified a positive association between hypertension and AR whereas in the studies by Hashimoto and Futamura^37^ and Hwang et al.^38^ the correlation between AR and high BP was negative, as was the association between AR and fasting plasma glucose (*P* < 0.05).

### Asthma and MetS

Ten studies evaluating the association between asthma and MetS were included in our review, and their overall results were similar. In the prospective cohort study by Nejatifar et al.^39^, the correlations of WC with asthmatic spirometry indices, DM, hypertriglyceridemia, and hypertension were significantly negative (*P* < 0.05). Brumpton et al.^40^ also conducted a prospective cohort study, which showed that MetS is a risk factor for incident asthma (adjusted OR 1.57, 95% CI 1.31–1.87). Similarly, an analysis of the 2007 to 2012 KNHANES data indicated that individuals with MetS had a significantly higher prevalence of asthma (adjusted OR 1.34, 95% CI 1.09–1.64)^41^. All seven of the reviewed studies concluded that a high WC is a significant risk factor for asthma, evidenced by the adjusted OR^39–45^.

## Discussion

The common pathophysiological features of AD, AR, and asthma, in which a Th2-predominant inflammatory response is the shared mechanism, account for their collectively forming the “atopic march”^46–48^. In previous studies demonstrating an inverse correlation between AD and MetS^6,27–29,31^, a proposed mechanism was significant histamine secretion from AD-induced mast cells, with effects on the skin including the dilation of the peripheral blood vessels, contributing in turn to a lower BP^49,50^. Cytokines such as IL-6 and TNF-α play a role in linking allergic diseases and metabolic syndrome. They interfere with insulin receptor substrate phosphorylation, reducing glucose uptake and increasing hepatic gluconeogenesis^51^. Additionally, adipokines like leptin and adiponectin mediate chronic inflammation and metabolic dysfunction^52^. Pro-inflammatory leptin promotes immune responses, while anti-inflammatory adiponectin, which is often reduced in metabolic syndrome and obesity, enhances insulin sensitivity. This imbalance may exacerbate systemic inflammation and contribute to metabolic disturbances.

Our study also showed that patients with AD were less likely to have hypertension and DM, although other studies found a positive correlation between AD and MetS^16,17,22,26^. The cytokines released in AD, including interleukin (IL)−17 and IL-22, both of which play a role in the chronic phase of AD^53^, were suggested to increase cardiovascular risks^54–56^, with their chronic inflammatory effects increasing the risk of MetS in AD patients^25,57,58^. The conflicting results obtained in our study vs. previous studies may reflect different genetic, socioeconomic, nutritional, and environmental factors. In addition, our study did not consider AD severity, which might explain the negative association between AD and hypertension or AD and DM.

Research on the relationship between metabolic syndrome and allergic diseases is limited in Asian countries. For example, recent studies from Japan and Taiwan suggest that obesity may increase the risk of asthma^59,60^. Another study in Japan has shown an association between obesity and allergic diseases but not metabolic syndrome^42^. A meta-analysis of Asian populations found moderate evidence linking obesity to atopic dermatitis^61^,consistent with a study demonstrating that BMI per age correlates with an increased risk of atopic dermatitis in Taiwanese and Vietnamese children but not in Japanese children^62^. Conversely, a study in Hong Kong found no link between obesity and asthma or atopy in Chinese children^63^. These findings highlight the diverse results across different populations, underscoring the need for further research to explore the relationship between metabolic syndrome and allergic diseases in greater depth.

Previous investigations examining the association between AR and MetS reported their negative association, including associations with central obesity^32,33^, hyperglycemia^37,38^, and hypertension^37,38^, consistent with our findings. While AR is dominated by Th2 cells, DM is predominantly associated Th1 cells, with their mutually exclusive actions resulting in the mutual suppression of their effects^38,64^. However, some studies have shown an increased prevalence of hypertension^32,35,36^, possibly due to atherosclerosis exacerbated by inflammatory mediators^54,65^ or hypoxia caused by nasal obstruction^66,67^. A retrospective study in Egypt^34^ reported a positive relationship between AR and dyslipidemia, possibly because the latter could tilt the immune response towards Th2 dominance^68,69^. The discrepant findings may be due to differences in the race and ethnicity of the study populations. For instance, obstructive sleep apnea, a suggested mechanism for the development of hypertension in AR, is less common in Asians than in the general US population, due to the lower body mass index of the former^54^.

Research on asthma and MetS has generally shown a positive correlation. Our study identified a positive relationship of asthma with all MetS components except high triglycerides. Adipose tissue in obese MetS patients produces pro-inflammatory cytokines such as IL-6, tumor necrosis factor-α, and leptin, which might contribute to airway inflammation^70^. Insulin resistance, a common feature of MetS, may also reduce glucose utilization and lead to skeletal muscle weakness, ultimately affecting airway function^71^. Additionally, in asthma patients the therapeutic use of high-dose steroids, which have mineral corticosteroid effects, may lead to sodium and water retention and therefore to hypertension^41^. Importantly, in our review, the direction of associations was largely consistent regardless of whether allergic diseases were defined as ‘ever diagnosed’ or ‘currently diagnosed’, suggesting that the choice of definition did not materially influence the overall findings. This clarification may help readers better interpret the variability in diagnostic criteria across studies.

The limitations of our study should be considered. First, because of the cross-sectional nature of the study, causal relationships between each allergic disease and MetS could not be defined. Second, potential confounders were included but other, unidentified factors may also have contributed to the identified associations. Third, because the KNHANES dataset defines allergic diseases as those ever diagnosed by a physician, we could not differentiate between current and past conditions. This may lead to an overestimation of disease prevalence or attenuation of associations, as some allergic diseases tend to resolve with age, particularly for conditions such as atopic dermatitis and pediatric asthma, which often remit with age. Fourth, this study is based on pre-collected data and therefore could not adjust for all potential influencing factors on allergic diseases, such as genetic predispositions and pollution. Fifth, since data collection relied primarily on self-reporting, the validity and reliability of several measurements used in the survey were limited, potentially leading to the misclassification of participants. In addition, in this study, the classification of residential area followed the KNHANES framework, in which all provinces outside the eight metropolitan cities were designated as rural. However, this categorization may not fully represent the heterogeneity within provinces, as large modern cities such as Changwon and Jeonju are included in the ‘rural’ category. This may result in partial misclassification and limit the accuracy of urban–rural comparisons. Also, this study classified hypertriglyceridemia based on triglyceride levels ≥ 150 mg/dL, excluding individuals taking lipid-lowering medications with lower levels. While this approach aligns with the NCEP-ATP III guidelines, it may have underestimated the prevalence of metabolic syndrome among treated individuals and could introduce bias in interpreting the association between metabolic syndrome and allergic diseases. Lastly, after reviewing previous research findings, we identified differences in how the diagnostic criteria for each allergic disease were established. We found that most studies applied the “ever diagnosed” criterion rather than the “currently diagnosed” criterion, which refers to having been diagnosed at a certain point or over a certain duration in the past. Nevertheless, no clear differences were observed in the direction of results, whether the association showed a positive or negative correlation, between “currently diagnosed” and “ever diagnosed” cases.

The strengths of our study lie in its utilization of data from a nationwide survey of a large population, which reduced the impact of selection bias. To our knowledge, this is the first study to investigate the association between allergic diseases and MetS components. Supported by the literature review, it thus offers a comprehensive perspective on the association between representative allergic diseases and MetS.

In summary, our study showed an inverse correlation between the prevalence of MetS and both AD and AR and a positive relationship between MetS and asthma. However, the conflicting outcomes regarding the relationship between allergic diseases and both MetS itself and each component of MetS reported in previous investigations remain to be clarified. Type 2 inflammatory pathways constitute the main player in allergic diseases, but elucidation of a connection to non-atopic conditions such as MetS is challenging due to the intricate and complex pathomechanisms involved, which hinder the identification of a unidirectional tendency. It is therefore not yet possible to draw definitive conclusions either on the associations between allergic diseases such as AD, AR, and asthma and MetS or on the underlying mechanisms; instead, further studies are needed.

## Supplementary Information

Below is the link to the electronic supplementary material.

Supplementary Material 1

## Data Availability

All relevant data are within the manuscript.
