# Supplementary material for: Associations between metabolic syndrome and allergic diseases a nationwide study in Korea and literature review
Source: Sci Rep. 2026 Mar 3;16:11889. doi: 10.1038/s41598-026-41559-3 (PMC13066066; doi:10.1038/s41598-026-41559-3)
Supplement: Supplementary file 1 — Supplementary Material 1 [file 41598_2026_41559_MOESM1_ESM.docx]

| **Supplement Table 1. General chraracteristics of the study population** | | | | | | | | | | | | | | | | | | | | | |
| --- | --- | --- | --- | --- | --- | --- | --- | --- | --- | --- | --- | --- | --- | --- | --- | --- | --- | --- | --- | --- | --- |
| **Variables** | |  | **Atopic Dermatitis** | | | | |  | **Allergic Rhinitis** | | | | |  | | | **Asthma** | | | | |
|  |  |  | **No** | | **Yes** | | **P-value** |  | **No** | | **Yes** | | **P-value** | |  | **No** | | | **Yes** | | **P-value** |
|  |  |  | **N** | **(%)** | **N** | **(%)** |  |  | **N** | **(%)** | **N** | **(%)** |  |  |  | **N** | | **(%)** | **N** | **(%)** |  |
| TOTAL | | 47,144 | 45,815 | (97.2) | 1,329 | (2.8) |  | 47,144 | 42,320 | (89.8) | 4,824 | (10.2) |  | | 47,144 | 45,972 | | (97.5) | 1,172 | (2.5) |  |
| **Metabolic Syndrome** | |  |  |  |  |  | <0.0001 |  |  |  |  |  | <0.0001 | |  |  | |  |  |  | <0.0001 |
|  | No | 33,433 | 32,318 | (70.5) | 1,115 | (3.3) |  | 33,433 | 29,585 | (88.5) | 3,848 | (11.5) |  | | 33,433 | 32,863 | | (98.3) | 570 | (1.7) |  |
|  | Yes | 13,711 | 13,497 | (98.4) | 214 | (1.6) |  | 13,711 | 12,735 | (92.9) | 976 | (7.1) |  | | 13,711 | 13,109 | | (95.6) | 602 | (4.4) |  |
| **Sex** |  |  |  |  |  |  | 0.0376 |  |  |  |  |  | <0.0001 | |  |  | |  |  |  | <0.0001 |
|  | Male | 22,146 | 21,559 | (97.4) | 587 | (2.7) |  | 22,146 | 20,332 | (91.8) | 1,814 | (8.2) |  | | 22,146 | 21,400 | | (96.6) | 746 | (3.4) |  |
|  | Female | 24,998 | 24,256 | (97.0) | 742 | (3.0) |  | 24,998 | 21,988 | (88.0) | 3,010 | (12.0) |  | | 24,998 | 24,572 | | (98.3) | 426 | (1.7) |  |
| **Age** |  |  |  |  |  |  | <0.0001 |  |  |  |  |  | <0.0001 | |  |  | |  |  |  | <0.0001 |
|  | 19-64 | 11,020 | 10,805 | (98.1) | 215 | (2.0) |  | 37,831 | 33,449 | (88.4) | 4,382 | (11.6) |  | | 11,020 | 10,765 | | (97.7) | 255 | (2.3) |  |
|  | 65- | 6,874 | 6,801 | (98.9) | 73 | (1.1) |  | 9,313 | 8,871 | (95.3) | 442 | (4.8) |  | | 6,874 | 6,322 | | (92.0) | 552 | (8.0) |  |
| **Region** | |  |  |  |  |  | 0.0002 |  |  |  |  |  | <0.0001 | |  |  | |  |  |  | 0.0444 |
|  | Rural | 27,733 | 26,885 | (96.9) | 848 | (3.1) |  | 27,733 | 24,607 | (88.7) | 3,126 | (11.3) |  | | 27,733 | 27,077 | | (97.6) | 656 | (2.4) |  |
|  | Urban | 19,411 | 18,930 | (97.5) | 481 | (2.5) |  | 19,411 | 17,713 | (91.3) | 1,698 | (8.8) |  | | 19,411 | 18,895 | | (97.3) | 516 | (2.7) |  |
| **Education Level** | |  |  |  |  |  | <0.0001 |  |  |  |  |  | <0.0001 | |  |  | |  |  |  | <0.0001 |
|  | Elementary school or lower | 8,562 | 8,468 | (98.9) | 94 | (1.1) |  | 8,562 | 8,172 | (95.4) | 390 | (4.6) |  | | 8,562 | 8,078 | | (94.4) | 484 | (5.7) |  |
|  | Middle school graduate | 4,723 | 4,650 | (98.5) | 73 | (1.6) |  | 4,723 | 4,416 | (93.5) | 307 | (6.5) |  | | 4,723 | 4,516 | | (95.6) | 207 | (4.4) |  |
|  | High school graduate, or college graduate or higher | 33,859 | 32,697 | (3.4) | 1,162 | (3.4) |  | 33,859 | 29,732 | (87.8) | 4,127 | (12.2) |  | | 33,859 | 33,378 | | (98.6) | 481 | (1.4) |  |
| **Income** | |  |  |  |  |  | <0.0001 |  |  |  |  |  | <0.0001 | |  |  | |  |  |  | <0.0001 |
|  | Low | 19,410 | 18,935 | (97.6) | 475 | (2.5) |  | 19,410 | 17,710 | (91.2) | 1,700 | (8.8) |  | | 19,410 | 18,695 | | (96.3) | 715 | (3.7) |  |
|  | High | 27,734 | 26,880 | (96.9) | 854 | (3.1) |  | 27,734 | 24,610 | (88.7) | 3,124 | (11.3) |  | | 27,734 | 27,277 | | (98.4) | 457 | (1.7) |  |
| **Occupation status** | |  |  |  |  |  | 0.0004 |  |  |  |  |  | 0.1323 | |  |  | |  |  |  | <0.0001 |
|  | Retired | 17,364 | 16,813 | (96.8) | 551 | (3.2) |  | 17,364 | 15,635 | (90.0) | 1,729 | (10.0) |  | | 17,364 | 16,705 | | (96.2) | 659 | (3.8) |  |
|  | Working | 29,780 | 29,002 | (97.4) | 778 | (2.6) |  | 29,780 | 26,685 | (89.6) | 3,095 | (10.4) |  | | 29,780 | 29,267 | | (98.3) | 513 | (1.7) |  |
| **Physical activity** | |  |  |  |  |  | 0.0131 |  |  |  |  |  | <0.0001 | |  |  | |  |  |  | <0.0001 |
|  | Other | 23,099 | 22,430 | (97.1) | 669 | (2.9) |  | 23,099 | 19,794 | (85.7) | 3,305 | (14.3) |  | | 23,099 | 22,449 | | (97.2) | 650 | (2.8) |  |
|  | Moderate intensity activity more than five times per week | 4,592 | 4,438 | (96.7) | 154 | (3.4) |  | 4,592 | 3,785 | (82.4) | 807 | (17.6) |  | | 4,592 | 4,510 | | (98.2) | 82 | (1.8) |  |
|  | Vigorous activity more than three times per week | 19,453 | 18,947 | (97.4) | 506 | (2.6) |  | 19,453 | 18,741 | (96.3) | 712 | (3.7) |  | | 19,453 | 19,013 | | (97.7) | 440 | (2.3) |  |
| **Smoking** | |  |  |  |  |  | 0.0455 |  |  |  |  |  | <0.0001 | |  |  | |  |  |  | 0.0645 |
|  | No | 37,188 | 36,169 | (97.3) | 1,019 | (2.7) |  | 37,188 | 33,162 | (89.2) | 4,026 | (10.8) |  | | 37,188 | 36,238 | | (97.5) | 950 | (2.6) |  |
|  | Yes (present) | 9,956 | 9,646 | (96.9) | 310 | (3.1) |  | 9,956 | 9,158 | (92.0) | 798 | (8.0) |  | | 9,956 | 9,734 | | (97.8) | 222 | (2.2) |  |
| **Drinking** | |  |  |  |  |  | <0.0001 |  |  |  |  |  | <0.0001 | |  |  | |  |  |  | <0.0001 |
|  | Not drinking for the last year | 8,284 | 8,104 | (97.8) | 180 | (2.2) |  | 8,284 | 7,485 | (90.4) | 799 | (9.7) |  | | 8,284 | 7,938 | | (95.8) | 346 | (4.2) |  |
|  | less than once a month | 9,958 | 9,670 | (97.1) | 288 | (2.9) |  | 9,958 | 8,901 | (89.4) | 1,057 | (10.6) |  | | 9,958 | 9,732 | | (97.7) | 226 | (2.3) |  |
|  | once a month | 5,422 | 5,250 | (96.8) | 172 | (3.2) |  | 5,422 | 4,822 | (88.9) | 600 | (11.1) |  | | 5,422 | 5,317 | | (98.1) | 105 | (1.9) |  |
|  | two to four times a month | 11,900 | 11,497 | (96.6) | 403 | (3.4) |  | 11,900 | 10,548 | (88.6) | 1,352 | (11.4) |  | | 11,900 | 11,691 | | (98.2) | 209 | (1.8) |  |
|  | Two to three times a week | 7,918 | 7,697 | (97.2) | 221 | (2.8) |  | 7,918 | 7,139 | (90.2) | 779 | (9.8) |  | | 7,918 | 7,755 | | (97.9) | 163 | (2.1) |  |
|  | more than four times a week | 3,662 | 3,597 | (98.2) | 65 | (1.8) |  | 3,662 | 3,425 | (93.5) | 237 | (6.5) |  | | 3,662 | 3,539 | | (96.6) | 123 | (3.4) |  |
| **Cancer** | |  |  |  |  |  | 0.0082 |  |  |  |  |  | 0.535 | |  |  | |  |  |  | 0.0676 |
|  | No | 46,506 | 45,184 | (97.2) | 1,322 | (2.8) |  | 46,506 | 41,752 | (89.8) | 4,754 | (10.2) |  | | 46,506 | 45,357 | | (97.5) | 1,149 | (2.5) |  |
|  | Yes | 638 | 631 | (98.9) | 7 | (1.1) |  | 638 | 568 | (89.0) | 70 | (11.0) |  | | 638 | 615 | | (96.4) | 23 | (3.6) |  |
